# Exploring Infertile Couples’ Decisions to Disclose Donor Conception to The Future Child 

**DOI:** 10.22074/ijfs.2020.44408

**Published:** 2020-10-12

**Authors:** Fatemeh Hadizadeh-Talasaz, Masoumeh Simbar, Robab Latifnejad Roudsari

**Affiliations:** 1 Department of Midwifery, Faculty of Medicine, Social Development and Health Promotion Research Centre, Gonabad University of Medical Sciences, Gonabad, Iran; 2 Midwifery and Reproductive Health Research Centre, Department of Midwifery and Reproductive Health, School of Nursing and Midwifery, Shahid Beheshti University of Medical Sciences, Tehran, Iran; 3 Nursing and Midwifery Care Research Centre, Mashhad University of Medical Sciences, Mashhad, Iran; 4 Department of Midwifery, School of Nursing and Midwifery, Mashhad University of Medical Sciences, Mashhad, Iran

**Keywords:** Child, Decision Making, Disclosure, Donor Conception, Infertility

## Abstract

**Background:**

Despite significant advances in reproductive technology, using donor assisted reproductive technology is a double-edged sword that has numerous challenges. One of the most challenging issues for couples is whether or not to disclose this information to donor offspring. This study, therefore, explored infertile couples’ decision to dis- close donor conception to their future child.

**Materials and Methods:**

This qualitative study was conducted using content analysis approach in 2012 in the Milad Infertility Centre, Mashhad, Iran. Data were collected through semi-structured interviews with 32 infertile persons including nine couples and 14 women who were selected by purposive sampling. Data were analysed by conven- tional qualitative content analysis adopted by Graneheim and Lundman using MAXQDA 2010 software.

**Results:**

Two categories were emerged: ‘not to disclose information to the child’ and ‘to disclose information to the child’. The first category consisted of three subcategories: 1. child support from probable harms; 2.to maintain healthy family relationships; and 3. lack of a compelling reason to disclose this information. The second category embraced four subcategories: 1. awareness of the others; 2. emergence of new living conditions; 3. appreciation for the donor; and 4. honesty among family members. The main reason for not disclosing information was to protect the child from probable harm.

**Conclusion:**

Although protecting children from possible harms was a major reason for infertile couples' secrecy, keeping this secret would not be always easy. Therefore, increasing public awareness about the donation process in order to change the beliefs of community and eliminate the infertile couples’ concerns would help them to overcome this problem. Additionally, long-term psychological counselling during and after the donation process is highly rec- ommended.

## Introduction

Infertility is a global reproductive health problem (1) that affects 8-12% of childbearing couples worldwide (2). Globally, approximately 48.5 million couples suffer from infertility (3). The experience of infertility can cause a wide range of social, psychological, physical and financial problems for couples (4-6). Despite considerable advances in assisted reproductive technology (ART) and new methods for becoming pregnant (7), which include donor conception, its use is compared to a double-edged sword that has many challenges for infertile couples. One of the most challenging issues of donor conception pertains to disclosure of this information to the child (8, 9). In other words, having a healthy baby does not end the challenges faced by the couples who undergo ART (10). Parents are confronted with many difficult questions that include how, what, when, and whether to disclose this information (11). In the past, reproductive endocrinologists advocated confidentiality and, prior to 1980, parents were advised to maintain secrecy regarding assisted reproductive donation procedures. However, the attitudes and approaches regarding these procedures have changed in recent decades and a sincere atmosphere has emerged among specialists due to lower levels of perceived stigma following the increased use of ART (10). Concerning the importance of disclosure to the child, countries such as Switzerland, New Zealand and the State of Victoria in Australia enacted laws on access by children to their origin of conception (12). Legislations in some countries have granted these children the right to be informed regarding their biological parents once the child reaches maturity (8, 10). Different countries have varying approaches in terms of matters of confidentiality. Although all forms of ART in Iran are approved by religious leaders, there is only a law for embryo donation and no laws exist for other donation methods. According to implementing regulations of the embryo donation law, the transfer of donated embryos is carried out in strictly confidential conditions. The documents and information on donors and recipients of donated embryos can only be obtained by order of the judicial authorities (13).

Studies around the world have reported different results regarding couples' views on disclosure of this information to the child. Some couples agreed to tell the child, whereas others have a negative opinion of this matter (10). In some studies, most couples who underwent fertilization via donated oocytes either chose to not disclose this information or were undecided (14-16). Conversely, in some other studies, the results were inconsistent. For example, in one study, 78% of donor oocyte recipients had decided to tell the child, 16% initiated the disclosure process, and 6% had planned for non-disclosure or were uncertain (16-18).

In Iran, few studies have reported the views of infertile couples in relation to the issue of disclosure. In a study conducted in Tabriz, most female respondents believed that it would be preferable for children born through surrogacy to not be told about the procedure (19). In a study in Tehran, most women also stated that they had no intention to disclose the gamete donation conception to their children (20).

We undertook this study because in recent years we have seen a sharp increase in the use of donated eggs (21) and, to the best of our knowledge, no qualitative research has been conducted in Iran regarding the decision to disclose this information to the donor children. This is the first Iranian study that has used the qualitative approach to explore decisions by infertile couples who underwent donor conception that pertained to whether they might disclose or not to disclose this information to their future children. It is hoped that the findings of this study will be useful for policy making, planning and providing services to infertile couples in Iran and other countries with similar social and cultural contexts.

## Materials and Methods

This qualitative study was conducted using the content analysis approach. Qualitative study, where participants' experiences are extracted, is appropriate for issues where information is limited and no prior research has been conducted (22).

The study was conducted in Mashhad, Iran in 2012. The participants consisted of 32 persons, which included nine infertile couples and 14 infertile women candidates for the following: donor eggs (11 persons), donor embryo (seven persons), donor egg and surrogacy (two persons), and surrogacy (three persons). The participants were purposively selected and interviewed.

The study population included all Iranian, Persian-speaking couples who referred to the Milad Infertility Centre with a diagnosis of infertility due to male, female, both, or unknown reasons. The participants were candidates for donor conception at the time of the study or beforehand and did not have biological children or stepchildren.

The sampling was purposive and we attempted to take into account the maximum variety of participants' choices in terms of age, education, economic status, cause of infertility, and duration of infertility and its treatment.

Data were collected using in-depth semi-structured interviews by the first author. Before starting the interview, each participant was informed about the purpose of the study and the study duration. Participants signed a written informed consent for study participation. The interview started with a general question about the purpose of the study (describe your experience with deciding to disclose the use of donation methods to your future child) and followed with the following questions: ‘What factors influenced your decision-making? How did these factors influence your decision making? What were your difficulties and obstacles in making this decision?’ Based on the participants’ responses, the interviewer asked additional questions or used probing questions to direct the interviews to elicit the participants’ experiences. Sampling continued without restriction in the number of participants until data saturation. A second or third interview was arranged to complete the data when the researcher faced gaps in the data during analysis and needed to ask new questions from particular participants. Each interview took between 30-120 minutes. Interviews with infertile couples were conducted separately; if there was a clear disagreement between their responses, a joint interview with the husband and wife was also held.

Data collection and analysis were carried out simultaneously by following the principles of conventional qualitative content analysis adopted by Graneheim and Lundman where coding and categorizing originate directly from the text (23). Conventional content analysis is usually used when the purpose of the study is to describe a phenomenon in which limited studies are available (24). Data were analysed in four stages. In the first stage, all recorded interviews were transcribed verbatim and considered as an analysis unit. In the second stage, by repeated reading of the transcripts, immersion in the data was performed to obtain a general insight. After repeated reading, the text was divided into sentences and paragraphs to identify the meaning units. In the third step, through process of reduction and condensation of meaning units, concepts or key ideas that were hidden within the meaning units, were coded. In the fourth step, similar codes were grouped into categories using MAXQDA 2010 software (25).

In order to increase the credibility of the study, variations in research participants, repeated reading of the interviews, long-term engagement with participants and the research environment during data collection and feedback from the participants (member check) were used. Dependability of the data was established through feedback from an external observer experienced in qualitative research.

To ensure the confirmability of the findings, the text of few interviews, codes and extracted categories were given to two researchers who were familiar with qualitative data analysis to confirm the accuracy of the process of data analysis. To examine transferability, two women who did not take part in the study, but had the similar profile with the participants, were asked to elaborate their experiences regarding the disclosure of the donor conception to the future child. What they explained was a reflexion of what researchers found in this study.

The study was approved by the Ethics Committees of Shahid Beheshti University of Medical Sciences, Tehran, Iran (Research Project Code: SBUMS. 8717). Prior to the interview, the purpose of the study was explained to the participants who subsequently provided their written informed consent. Participants were assured that their information would be kept confidential. All participants were allowed to withdraw from the study at any time without any change in their care plan. Prior to the interview, the permission of participants for recording their voice was taken; just one participant disagreed, so notes were taken during her interview.

## Results

The age range of the women who participated in the study was 23-41 years, the age range of the men who participated was 26-40 years, the duration of marriage ranged from 7-14 years, and the duration of infertility treatment with donation methods ranged from 1-4 years. The education in women ranged from elementary to bachelor’s degree and, in men, from diploma to postgraduate.

Couple’s decisions about disclosure of the use of donor conception to the child included two categories including not to disclose to the child and to disclose to the child (Table 1).

**Table 1:** Categories and sub-categories emerged from the data


Sub-Categories	Categories

Child support from possible harms To maintain healthy fam­ily relationships No compelling reasons for disclosure	Not to disclose information to the child
Awareness of others Emergence of new living conditionsAppreciation for the donor Honesty among family members	To disclose information to the child


### Not to disclose information to the child

Data analysis demonstrated that most participants decided not to disclose the donation process to the child. The category of ‘Not to disclose information to the child’ appeared from three subcategories of ‘child support from probable harm’, ‘to maintain healthy family relationships’, and ‘no compelling reasons to disclose’, which are discussed in detail. The most commonly cited sub-category was ‘child support from probable harms’.

### Child support from probable harms

Most participants stated that they were mostly concerned about the child poor of acceptance and his possible reaction. The child’s limited understanding of the current normal life situation and making him aware of the different aspects of birth would make it very difficult for the child. Therefore, disclosure would be a sensitive issue that could cause emotional and psychological trauma. Secrecy about the origin of the child, infliction of unnecessary emotional burden on the child and the possible harms and confusion could be prevented. One participant stated:

“Major trauma will be imposed on the child when somebody tells him that his current parents are not his genetic parents - even if this situation would happen to me at this age, of course I would be upset and would be generally confused, you know, definitely it is much harder for a young child " (40-year-old male, candidate for embryo donation).

Aside from the social stigma that exists with pregnancy from donation procedures, disclosure of the origin of pregnancy for the reason that the child is seen as different from other children would result in isolation, loneliness, fear, and stress. The child would be haunted by the question, ‘Who do I belong to? Are my real parents good or bad?’ Awareness of the issue may cause the child to feel shame and his sense of self-worth might be damaged, which would affect the child’s self-esteem and feelings of identity, as one participant expressed:

“Once the child knows, he will become isolated and when he is exposed to the society, he will have the impression that he is different from the others and would feel isolated” (31-year-old female, candidate for oocyte donation).

Despite this issue, some couples are concerned that the child will unexpectedly discover the information regarding his identity. One respondent stated:

"Even if we have children, our problems are not over and we still have to worry about the child realizing the truth) "34-year-old male, candidate for egg donation).

### To maintain healthy family relationships

Couples are always concerned about the destruction of family relationships, in particular the relationships between parents and the child following disclosure of this information. They are concerned that disclosure might lead to low sense of belonging and family dependency in the child. As a consequence, it causes a change in perception towards the parents and, ultimately, the child’s rejection of the parents would happen.

“I’m telling you, once the child knows about the issue, his thoughts will be preoccupied unconstructively and he might say, ‘my parents are lying to me and I’m not their child’ ” (36-year-old female, candidate for egg donation).

### No compelling reasons to disclose

Some of the participants stated that there is no need to make the child’s life complicated with confusing and stressful information. Telling the truth to the child would not benefit the child because he cannot do anything about it. On the other side, the genetic relationships between the child and the couple would result in some ownership to the couples and, through pregnancy and exchange of blood and nutrients between the mother and the foetus, the biologic relationships and the role of the mother is maintained. Thus, disclosure is not considered to be vital. Breastfeeding can also help maintain this role. One participant stated:

”It is not necessary to disclose this information. The child is mine, the sperm is mine and the child will develop inside my wife’s uterus. After birth, my wife will breastfeed the child” (36-year-old male, candidate for egg donation).

There was an initial agreement between the couple's decisions about not disclosing this information to the child. They both decided not to inform the child about the donation procedure.

### To disclose information to the child

Regarding the category of to disclose this information to the child’, data analysis revealed that only a few couples reported the intent to disclose. This category was derived from four subcategories: ‘awareness of the others’, ‘emergence of new living conditions’, ‘appreciation for the donor’ and ‘honesty among family members’. ‘Awareness of others’ was the subcategory most often mentioned by the participants.

### Awareness of others

Awareness of the others plays an important role in the couple's decision. When others are aware of the issue, it becomes important that parents tell the child the truth in order to prevent accidental disclosure by others. Being told the truth by others would result in the child's feelings of distrust toward her/his parents. One respondent commented:

In my opinion, it is much better that we will tell the truth ourselves because when others know about this issue, the child must also know" (43-year-old female, candidate for surrogacy).

### Emergence of new living conditions

Couples who desired secrecy commented that the emergence of a new life situation would cause them to re-evaluate their decisions because disclosure varies depending on the future situation of the community and, with passage of time, peoples’ knowledge and awareness of these procedures will increase. This would pave the way for a community easier acceptance and decreased exposure of the child to social stigma. Also, in case the same problems exists for future offspring and disclosure is necessary to protect the child, the couple's decision will change and there is a greater possibility of telling the truth to the child at the appropriate time. One participant explained this idea in the following way:

"In cases where the same problem exists for the child, I would say the fact. I will only disclose the truth when my child becomes wiser, and can identify and be able to understand the situation better" (41-year-old female, candidate for oocyte donation and surrogacy).

### Appreciation for the donor

Regarding the use of familiar donors, disclosure to the child at an appropriate age for the purpose of appreciating the donor is predictable, as one participant stated:

"It is much better that the child be told that her auntie is also his rightful mother, and should know that she/he has some duties towards her and is entitled to many rights" (31-year-female, candidate for surrogacy).

### Honesty among family members

One of the participants explained that she made use of ethical reasons in making her decision to disclose, and the principle of honesty and the desire for honest and transparent relationships between family members. Although parents are known to be honest; however, most are only willing to partially tell the child about the truth about surrogacy, but not regarding egg donation. This indicates that the issue of absence of genetic relationships is more sensitive than surrogacy. One participant expressed this idea:

"I think that at least I can tell my child about surrogacy and I think it is easier since telling a lie is very difficult for me to do. I hate to lie and I always think of the consequences of lying" (39-year-old female, candidate for oocyte donation and surrogacy).

Most participants, from both urban and rural areas, decided to keep the use of donor conception secret. The results of this study also showed that among the types of donor conception, surrogate candidates were more likely to disclose the donation method to the child.

There was an agreement between the couple's decisions to disclose information to the child, and they both decided to inform the child about the donation procedure under certain circumstances.

## Discussion

The results of the present study indicated that most couples decided not to disclose the use of donor conception to their future child. This finding, when compared with studies conducted worldwide, indicated that levels of disclosure to the child in Iran are much lower. Despite general recommendations to parents in relation to disclosure of the donor conception to future offspring (16, 26), results of the studies conducted worldwide about disclosing the origin of pregnancy to the child yielded a wide range of results. A survey of 111 recipient couples that used donor eggs or sperm showed that the majority of participants planned for disclosure and some had begun the disclosure process. Only a few planned for non-disclosure or were not certain. (18). The results of a study in Spain (2014) showed that most participants intended to disclose donor conception to the child, whereas a few participants did not intend to disclose this information or had not yet decided at the time of completing the questionnaire (12). In some studies, most couples who conceived through oocyte donation decided not to disclose this information or were uncertain (14-16).

According our study, the main reason for non-disclosure to the child was to protect the child from probable harms. In some studies, protection of the child from possible harms, including psychological and moral harm, was the most common explanation (27). In a study conducted in Northern California, parents who did not disclose this information believed that nondisclosure to the child protected the child from unnecessary psychological pressure, avoided potential harm and confusion as well as the feeling of isolation (28). Studies that compared the long-term consequences of disclosure and non-disclosure by families have found that there is no difference in child welfare or the parent-child relationships (29-31). In contrast, some studies believe that not telling the truth may increase the child's psychological problems (32).

Another reason expressed by the current study participants for non-disclosure was to support and maintain family relationships. In a study, the most important motivation by couples toward non-disclosure of the donor conception to the child was fear of rejection by the family, social environment and/or the child (33). In some studies, the existence of stigma due to donation methods from the causes of secrecy has been expressed. In some studies, the stigma surrounded the issue of donor conception has been mentioned as a reason for secrecy (11).

Another reason stated by the current study participants for not disclosing was lack of compelling reasons for disclosure. This finding of this study is congruent with another study which found that many mothers decide not to disclose the issue of donor conception to the offspring, as they feel that there is no need to tell the child. For these mothers, genetics were far less important than parenting (10).

According to the findings of this study, awareness of others about this issue was the most common reason given by the couples who intended to disclose donation methods to the future child. There exists a clear relationship between disclosure and non-disclosure in relation to the child and society. Couples who opted for non-disclosure to the child were obviously more secretive towards the society, while couples who intended to disclose this information were more truthful on disclosing it to families and friends. Since the social environment may be aware of the problem of infertility and gamete donation, parents are always concerned about others disclosing this fact to the child. In one study, parents who decided to disclose donor conception expressed concern about accidental disclosure of this subject from someone other than the parents (28). A study in Iran assessed infertile couples’ decisions in relation to disclosure of donor conception to others. The results indicated that couples who chose not to disclose this information to others stressed the idea of child protection from accidental disclosure as it could affect the child-parent relationship and create a lack of trust about the parents (13).

Another reason mentioned by this study’s participants for disclosure was emergence of new living conditions. Parents who opted not to disclose stated that new living conditions might lead them to reconsider their decision (34).

Although the results of this study indicated that another reason for disclosure was appreciation for the donor, which was the case for familiar donors, differing results have been reported. Another study reported no significant difference between known and unknown oocyte recipients regarding disclosure to the future child (35).

In this study, another reason for disclosure was honesty among family members. This finding was consistent with the results from other studies (10, 11, 28, 33).

The child's right to know has been mentioned as an important factor for disclosure (11) in worldwide research studies; however, in the present study, this was not mentioned by any of the participants.

The fear of disclosure remains a difficult question: ‘When the child realizes the truth, does he or she still accept us as his or her parents?

Based on the results of current study, non-disclosure is accompanied by stress. Couples’ concern about disclosure remains as a hard question: When the child realizes the truth, does he/ she still accept couple as his or her parents? Worries and apprehension can also harm the sexual and emotional relationships of the couple (36). However, the results of this study also showed that disclosing information to the child may be associated with stress and negative effects on the couple and the child. According to one study, in some people, disclosure of the fertility conditions was like a double-edged sword that put additional pressure on them (37). In such circumstances, providing counselling can help them to cope better with their stressful situation and come to terms with their experiences (38). They should also be encouraged to adopt adaptive coping strategies in order to enhance their self-empowerment and to achieve a sense of personal wholeness by merging the bio-psycho-social perspectives (39).

The findings of this study showed that among the three types of donation, those who were candidates for surrogacy were more likely to disclose the truth to the child. Some candidates for a traditional surrogacy decided to disclose the use of the surrogate mother to the child, but not disclose the use of the surrogate mother’s egg. This might be due to decreased social stigma about surrogacy. This was consistent with the findings reported by Readings et al. (10). Research have shown that the presence or lack of biological communication with the child has a profound effect on the disclosure process in parents (40).

The strengths of this study include the use of a qualitative approach to directly reflect participants’ responses. In addition, this study was conducted in a referral centre admitting patients with different socio-cultural backgrounds. The information was obtained from couples rather than only women.

One of the limitations of this study was the lack of cooperation of some participants, which was due to the sensitivity of the issue of donation conception and its stigma.

In this research, the couples’ decisions to disclose information to the child was evaluated. However, conducting further studies for long-term assessments would be beneficial because the reported sentiments of the couples in relation to disclosure do not always reflect their future behaviour, and the decision to disclose might be different from the actual disclosure.

## Conclusion

The results of the present study indicated that most couples decided not to disclose the use of a donation procedure to their future child in order to protect the child from possible harms. The results of this study were somewhat different from those in other countries. The rate of disclosure to the child in Iran is very low, which might be due to the stigma of using donation methods in Iranian culture.

Therefore, we recommend interventions to change public perceptions to reduce this stigma and present fertility donation methods in a natural way to resolve the infertile couples’ concerns. Also, since the use of donor conception and childbirth does not end couples' concerns about disclosure, long-term counselling is recommended for these couples.
